# Enhancement of poly(3-hydroxybutyrate-*co*-3-hydroxyvalerate) accumulation in *Arxula adeninivorans* by stabilization of production

**DOI:** 10.1186/s12934-017-0751-4

**Published:** 2017-08-17

**Authors:** Mateusz Biernacki, Marek Marzec, Thomas Roick, Reinhard Pätz, Kim Baronian, Rüdiger Bode, Gotthard Kunze

**Affiliations:** 1 Leibniz Institute of Plant Genetics and Crop Plant Research (IPK), Corrensstr. 3, 06466 Gatersleben, Saxony-Anhalt Germany; 2 Jäckering Mühlen-und Nährmittelwerke GmbH, Vorsterhauser Weg 46, 59007 Hamm, Germany; 3 Division Bioprocess Technology, University of Applied Sciences, Bernburger Str. 55, 06366 Köthen, Germany; 4 School of Biological Sciences, University of Canterbury, Private Bag 4800, Christchurch, New Zealand; 5 Institute of Microbiology, University of Greifswald, Jahnstr. 15, 17487 Greifswald, Germany; 6 Faculty of Biology and Environmental Protection, University of Silesia, Jagiellonska, 28, 40-032 Katowice, Poland

**Keywords:** Polyhydroxyalkanoates, PHB-V, *Arxula adeninivorans*, Phasin

## Abstract

**Background:**

In recent years the production of biobased biodegradable plastics has been of interest of researchers partly due to the accumulation of non-biodegradable plastics in the environment and to the opportunity for new applications. Commonly investigated are the polyhydroxyalkanoates (PHAs) poly(hydroxybutyrate) and poly(hydroxybutyrate-*co*-hydroxyvalerate) (PHB-V). The latter has the advantage of being tougher and less brittle. The production of these polymers in bacteria is well established but production in yeast may have some advantages, e.g. the ability to use a broad spectrum of industrial by-products as a carbon sources.

**Results:**

In this study we increased the synthesis of PHB-V in the non-conventional yeast *Arxula adeninivorans* by stabilization of polymer accumulation via genetic modification and optimization of culture conditions. An *A. adeninivorans* strain with overexpressed PHA pathway genes for β-ketothiolase, acetoacetyl-CoA reductase, PHAs synthase and the *phasin* gene was able to accumulate an unexpectedly high level of polymer. It was found that an optimized strain cultivated in a shaking incubator is able to produce up to 52.1% of the DCW of PHB-V (10.8 g L^−1^) with 12.3%mol of PHV fraction. Although further optimization of cultivation conditions in a fed-batch bioreactor led to lower polymer content (15.3% of the DCW of PHB-V), the PHV fraction and total polymer level increased to 23.1%mol and 11.6 g L^−1^ respectively. Additionally, analysis of the product revealed that the polymer has a very low average molecular mass and unexpected melting and glass transition temperatures.

**Conclusions:**

This study indicates a potential of use for the non-conventional yeast, *A. adeninivorans,* as an efficient producer of polyhydroxyalkanoates.

## Background

Currently plastic products cause many problems with disposal and recycling is not sufficient to prevent accumulation. One of the solutions is the production of biodegradable polymers made from renewable substrates. The polyhydroxyalkanoates (PHA) polymers, which are naturally produced by bacteria as a storage material [1] are a well-known examples. These polymers may be modified for a variety of applications from packaging and agriculture through to medical implants and drug delivery devices [2]. PHA may be composed of more than 150 different monomers which give them a variety of physical and chemical properties [3]. The most well-known PHA, poly(hydroxybutyrate) (PHB), has some disadvantages, e.g. it is brittle, stiff and highly crystalline [4]. In contrast, poly(hydroxybutyrate-*co*-hydroxyvalerate) (PHB-V) has better flexibility, toughness and has lower melting and glass transition temperatures, depending on PHV content [5].

Synthesis of PHB-V is carried out by at least three enzymes: β-ketothiolase, (*R*)-specific NADPH-dependent acetoacetyl-CoA reductase and PHA synthase [6]. This pathway has been primarily investigated in bacteria, e.g. *Cupriavidus necator* for PHB-V production with range of accumulation level between 50 and 90% of DCW containing up to 24% PHV [1, 7]. Additionally yeast and plants have been employed for polymer production [8, 9]. Nevertheless, the entire industrial production of PHB-V is currently based on bacteria, which may be associated with certain problems such as phage contamination of *Escherichia coli* process [10] or possible presence of lipopolysaccharides in the product, which excludes its use in medical applications [11]. On the other hand yeast have a higher contamination resistance, broad substrate spectrum including industrial by-products and cultivation may be carried out in harsh environments, e.g. acidic or high sugar concentration [10]. The majority of yeast studies have been conducted using baker’s yeast (*Saccharomyces cerevisiae),* however, the obtained results were insufficient to compete with bacterial systems. Kocharin et al. [12] showed that a *S. cerevisiae* strain harbouring PHA pathway genes and additional genes for engineering acetyl-CoA metabolism is able to accumulate 0.25 g L^−1^ of PHB. In another study, overexpression of targeted PHA synthase to peroxisomes in *S. cerevisiae*, lead to the synthesis of up to 7% of DCW polymer composed of C_4_–C_8_ monomers [13]. Some non-conventional yeast also exhibit PHA synthesis, for example, *Kloeckera* spp. accumulated 7.03% DCW PHB-V [14] and *Pichia pastoris* grown on oleic acid was able to produce 1% of medium-chain-length PHA [15]. Recently Li et al. [16] presented a genetically engineered *Yarrowia lipolytica,* which accumulated PHB up to 10.2% DCW (7.35 g L^−1^) when grown on glucose and acetate, which is the highest level of PHA accumulation in yeast reported to date. However, production strategies in yeast have concentrated only on direct PHA synthesis and optimization of metabolism, and not on stabilization of accumulation. Phasins are a group of low-molecular-weight proteins with amphiphilic properties. In bacteria, where these proteins were originally found, they play a regulatory and stability role during PHA synthesis and cell division [17]. Moreover, *C. necator* phasin *PhaP1* gene, unexpectedly decreased cells stress when overexpressed in a non-phasin strain of *E. coli* [18].

Another non-conventional yeast, *Arxula adeninivorans,* was previously used for PHB-V production by Terentiev et al. who described an *Arxula* strain harbouring PHA pathway genes from *C. necator* which accumulated 0.019% PHB and 2.2% PHV using controllable ethanol fed-batch fermentation [19]. Since then the *A. adeninivorans* expression platform has been optimized and successfully used for production of a number of recombinant proteins [20–23]. Based on the improved platform, novel genetic modification techniques and codon optimization gene synthesis, it was proposed that it would be possible to improve PHB-V production in *Arxula*. Moreover *Arxula* does not have endogenous genes responsible for intracellular PHA degradation and because of its oleaginous character, has a high concentration of CoA and NADPH reduction power, which are necessary for PHA synthesis. Finally, the groundwork for this study was our previous research in which demonstrated that *A. adeninivorans,* with overexpressed *thl* thiolase from *Clostridium acetobutylicum* and *phaB* reductase from *C. necator,* is able to secret enantiomerically pure (*R*)-3-HB [24].

In present study we describe the optimized production and stable accumulation of PHB-V copolymer by overexpression of PHA pathways and *phasin* genes in the yeast, *A. adeninivorans.*

## Methods

### Strains and cultivation condition

*Escherichia coli* XL1 Blue [*recA1 endA1 gyrA96 thi*-*1 hsdR17 supE44 relA1 lac* [F´ *proAB lacI* ^*q*^ *Z∆M15* Tn*10* (Tet^r^)]], obtained from Invitrogen, was used for cloning experiments and plasmid isolation. Luria–Bertani (LB—Sigma, USA) supplemented with 100 mg L^−1^ ampicillin, 50 mg L^−1^ chloramphenicol or 50 mg L^−1^ kanamycin was used as a growth medium.

The wild-type strain, *A. adeninivorans* LS3, originally isolated from wood hydrolysate in Siberia and deposited as *A. adeninivorans* SBUG 724 in the strain collection of the Department of Biology of the University of Greifswald [25] was used as a control strain. The auxotrophic mutant, *A. adeninivorans* G1216 [*aleu2 ALEU2::aade2*] [26] and double auxotrophic mutant, MS1006 [*aleu2 atrp1::ALEU2 aade2::ALEU2*] [27] were used as recipient strains. All strains were cultivated at 30 °C, 180 rpm in 50 mL of broth in a 100 mL flask. The medium was either a selective yeast minimal medium supplemented with 20 g L^−1^ glucose and 43 mM NaNO_3_ (YMM-glc-NO_3_) [28, 29] or a non-selective yeast complex medium containing 20 g L^−1^ glucose (YPD).

### Co-substrate feeding

To check an influence of different co-substrates on polymer and copolymer synthesis, several of C-sources were trialled. Different concentrations of ethanol/1-propanol/propionic acid/valeric acid/sodium propionate were added to cultures after 48 and 96 h of cultivation.

### Fed-batch cultivation

Fed-batch cultures were performed in a 5-L bioreactor (Sartorius, Germany) with conditions set to optimize growth and PHB/PHB-V production. The temperature was maintained at 30 °C and a pH of 6.0 was maintained by the addition of 2.5 M NaOH or 1 M H_2_SO_4_. The level of oxygen was varied and maintained by the stirring rate. The culture was started in modified YPD followed by the addition of glucose or ethanol/1-propanol (1:1 v/v) and nitrogen to prevent C-source depletion and maintain the metabolism of the organism. Controlled addition of a silicone-based anti-foam agent (Strunktol SB 304, Schill+Seilacher GmbH, Germany) was employed to prevent foaming.

### Plasmid construction

#### Xplor2.4

The open reading frames (ORFs) of the bacterial genes were synthesized by GeneArt (Life Technologies) using optimized codon usage. All of the ORFs were inserted into pBS-TEF1-PHO5-SA vector with a pair of restriction sites to obtain expression modules containing the *A. adeninivorans* derived *TEF1* strong constitutive promoter and the *S. cerevisiae PHO5* terminator [30]. A set of primers (Table 1) and the above plasmids were used to amplify using PCR, the DNA fragments with an ORF, promoter, terminator and additional restriction sites. A multiple-step cloning procedure was used for the construction of the final expression plasmids based on Xplor2.4 system [26]. The TEF1-phaB-PHO5 fragment flanked by 5′-*Bsi*WI/*Spe*I and 3′-*Mlu*I/*Sac*II sites was introduced into the basic vector to create Xplor2.4-TEF1-phaB-PHO5. Subsequently, the expression module containing the *TEF1* promoter, *PHO5* terminator, one of the two *ß*-*ketothiolase* genes (*thl, bktB*) and 5′-*Spe*I and 3′-*Bsi*WI restriction sites was cloned into the vector to generate Xplor2.4-TEF1-thl/bktB-PHO5-TEF1-phaB-PHO5 plasmids. Next, the expression module TEF1-phaC-PHO5 with *PHAs synthase* gene and 5′-*Mlu*I and 3′-*Sac*II restriction sites was cloned into the above vector to obtain the final expression plasmid Xplor2.4-TEF1-thl/bktB-PHO5-TEF1-phaB-PHO5-TEF1-phaC-PHO5. Additionally, the expression module TEF1-phaP1-PHO5 with the *phasin* gene was inserted using 5′-*Sal*I and 3′-*Apa*I sites (originating from the basic pBS-TEF1-PHO5-SA vector) to generate the Xplor2.4-TEF1-thl/bktB-PHO5-TEF1-phaB-PHO5-TEF1-phaC-PHO5-TEF1-phaP1-PHO5 plasmids.

**Table 1:** Oligonucleotide primers and *A. adeninivorans* strains used in this work

Designation	Oligonucleotide sequence	Source
TEF1_SpeI	TATA*ACTAGT*TAGTAGCGCTAATCTATAATCAG	Eurofins genomics
PHO5_BsiWI	CGGA*CGTACG*AGCTTGCATGCCTGCAGA	
TEF1_MluI	TGACT*ACGCGT*CTCGACTTCAATCTATAATCAGTC	
PHO5_SacII	TATA*CCGCGG*CGGCCCCAGCTTGCATGCCTGCAGA	
TEF1_SpeI_BsiWI	TAT*ACTAGT*ACTT*CGTACG*CTCGACTTCAATCTATAATCAGTC	
PHO5_SacII_MluI	GGAT*CCGCGG*CCGA*ACGCGT*AGCTTGCATGCCTGCAGATTTTAATC	
Complete strain name		
AAG_thl	G1216/YIC104-thl-phaB-phaC	This study
AAG_thlp	G1216/YIC104-thl-phaB-phaC-phaP1	
AAG_bktBp	G1216/YIC104-bktB-phaB-phaC-phaP1	
AAMS_thlp	MS1006/YIC104-thl-phaB-phaC-phaP1-YRC102-thl-phaB-phaC-phaP1	
AAMS_bktBp	MS1006/YIC104-bktB-phaB-phaC-phaP1-YRC102-bktB-phaB-phaC-phaP1	

Italic letters indicate restriction sites

**Fig. 1:**
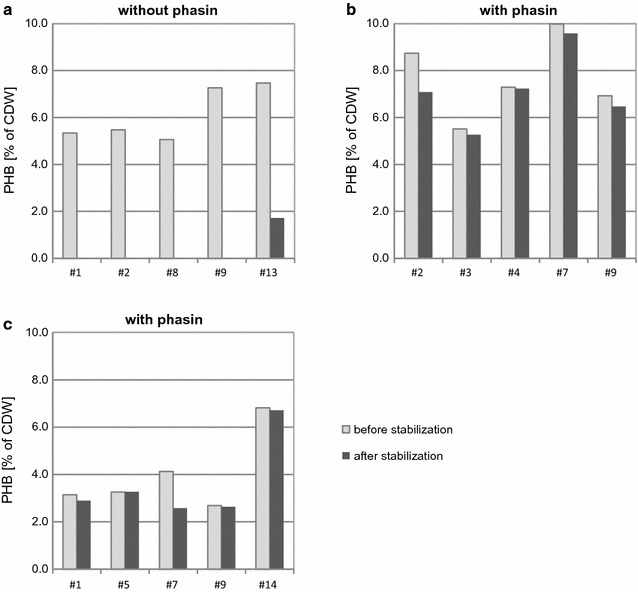
Influence of the yeast stabilization procedure and the overexpression of *phasin* gene on PHA production. The five transformants with the highest polymer content are shown for each strain and are marked as follows: **a** AAG_thl; **b** AAG_thlp; **c** AAG_bktBp. Only PHB was detected

All variants of the final plasmids were linearized with *Asc*I or *Sbf*I restriction enzymes and separately transformed into the auxotrophic and double auxotrophic strains, *A. adeninivorans* G1216 and MS1006 (Fig. 1). The latter strain’s growth medium was supplemented with 20 mg L^−1^ tryptophan to maintain growth after transformation (ATRP1 marker not yet complemented).

#### Xplor2.2

A similar multiple-step cloning procedure was used to prepare Xplor2.2-TEF1-thl/bktB-PHO5-TEF1-phaB-PHO5-TEF1-phaC-PHO5-TEF1-PhaP1-PHO5 plasmids. The Xplor2.2 expression platform is similar to Xplor2.4 with the only difference being that the auxotrophic marker is tryptophan instead of adenine [31].

Both variants of the plasmid, were linearized with *Asc*I or *Sbf*I restriction enzymes and separately transformed as previously described [31] into the auxotrophic strains containing the same expression module previously described to form *A. adeninivorans* MS1006/YIC104-thl-phaB-phaC-phaP1 and MS1006/YIC104-bktB-phaB-phaC-phaP1.

Stabilization of the yeast transformants was performed by passaging on selective and non-selective media as described by Klabunde et al. [32]. Plasmid DNA isolation and DNA restrictions were performed as described by Wartmann et al. [33].

### Gas chromatography/mass spectrometry (GC/MS) analysis

Gas chromatography/mass spectrometry analysis was used to determine the concentration of PHA in dried cells. 3–4 mg of lyophilized cells was subjected to propanolysis as described by Riis and Mai [34] but with modification. 1 mL 1,2-DCE and 1 mL n-propanol-HCl solution (4:1 v/v) was added to cells and incubated at 90 °C for 4 h. After cooling to room temperature the reaction mixture was extracted with 2 mL of triple distilled H_2_O and the lower organic phase was taken for analysis in a Clarus^®^ 680 GC combined with Clarus^®^ SQ 8 S MS (PerkinElmer, USA) equipped with Elite-624 column (PerkinElmer, 30 m × 0.5 mm, 1.4 µm). The GC program was set up as follows: initial temperature 80 °C for 5 min, ramping at 10 °C min^−1^ to 235 °C and held for 10 min.

Commercial PHB-V (Sigma-Aldrich, 12%mol of PHV) was used to construct a standard curve. The analysis was conducted in triplicate and results were analyzed by TurboMass 6.1 software.

### Polymer extraction and analysis

Poly(3-hydroxy)butyrate *co*-polymer produced by *Arxula* was isolated by solvent extraction method [35] with further modification. Yeast were harvested, washed twice with water and processed in a French Press. 30 mg L^−1^ of trypsin was added to the disrupted cell suspension and incubated in 37 °C for 1 h. The resulting cell debris was freeze-dried overnight and 5 g of lyophilized material was boiled for 6 h in 100 mL of chloroform. The excess of solvent was evaporated making the solution viscous which was then mixed dropwise with 10-times volume cold ethanol to precipitate final product. Finally the polymer was collected on a paper filter (2.7 µm, Whatman, USA) and dried in 55 °C.

Purity assessment was carried out by GC/MS. Gas permeation chromatography (GPC), thermoanalysis (DSC) and thermogravimetry (TGA) was performed by KIMW Prüf- und Analyse GmbH (Lüdenscheid, Germany) and mass spectrometry (TOF–SIMS analysis) by OFG—Analytik (Münster, Germany).

### Cell imaging

Imaging of PHA granules inside *Arxula* cells was performed using BODIPY 493/503 (Thermo Fisher, USA), a green fluorescent agent, which binds to neutral lipids and similar compounds. It was shown to be more suitable for PHA staining than Nile Red [36]. The cell material for microscopic analysis was collected after 240 h and twice washed with distilled water. 200 µL of cells suspension was mixed with 20 µL of 0.01% solution of BODIPY 493/503 in DMSO and incubated at room temperature for 5 min. The suspension was then centrifuged and resuspended in an equal volume of distilled water. Freshly prepared material was then analyzed using a confocal laser-scanning microscope (Zeiss LSM 780; Zeiss, Jena, Germany). The BODIPY signal was detected using an argon 488-laser equipped with a 560–615 nm band pass filter.

To enable ultrastructural examination using transmission electron microscopy (TEM), high pressure freezing followed by freeze substitution and resin infiltration were performed according to the method described by Daghma et al. [37]. Ultrathin sections of ~70 nm thickness were prepared as described previously [38] and visualised with a Tecnai Sphera G2 transmission electron microscope (FEI Company, Eindhoven, The Netherlands) set at 120 kV.

### Statistics

Two cultures were grown as independent experiments and GC/MS analysis was performed in triplicate. The final results are average values of the data.

## Results

### PHA shaking flask screening

Overexpression of *ß*-*ketothiolase* gene (origins from *C. acetobutylicum* or *C. necator* H16) and *acetoacetyl*-*CoA reductase* and *PHA synthase* genes from *C. necator* H16 in *A. adeninivorans* led to synthesis of PHA by this yeast. Additionally, the *phasin* gene from *C. necator* H16, which has been reported to be a stabilizer of PHA production [41], was overexpressed. The plasmids Xplor2.4-thl-phaB-phaC and Xplor2.2/Xplor2.4-thl/bktB-phaB-phaC-phaP1 were created with each containing all three production-genes and an additional gene to stabilize synthesis and flanked with the strong constitutive *TEF1* promoter and *PHO5* terminator. The endogenous auxotrophic AADE2 and/or ATRP1 markers, which restore adenine or tryptophan synthesis pathways in *A. adeninivorans* respectively, were used to select positive transformants. After linearization with *Asc*I (YRC—homologous recombination) or *Sbf*I (YIC—non-homologous recombination) plasmids were transformed into the corresponding auxotrophic mutants, *A. adeninivorans* G1216 or MS1006 and selected on media that does not contain adenine and/or tryptophan (Table 2).

**Table 2:** Overexpressed genes for PHA production

Gene		Accession No.	Organism
β-Ketothiolase	*thl*	LT608130	*C. acetobutylicum* ATCC 824
	*bktB*	PRJEB20372	*C. necator* H16
Acetoacetyl-CoA reductase	*phaB*	LT608132	*C. necator* H16
PHA synthase	*phaC*	PRJEB20372	*C. necator* H16
Phasin	*phaP1*	PRJEB20372	*C. necator* H16

The constructed strains used in this article are the result of overexpression of one of the *thiolase* genes, *phaB* reductase gene, *phaC* synthase gene and/or the *phaP1* phasin gene. All the ORFs were synthesized by codon optimization method

#### Cells without phasin gene

As the first screening procedure, strain AAG_thl was tested for PHA production. Before stabilization, measurements of PHA revealed that most of transformants were able to produce PHB after 96 h, with the highest polymer content 7.47% of DCW. On the other hand after stabilization only one of the transformants retained PHB production ability with 1.72% of DCW after 96 h which is only 23.1% of the initial production. The aim of the stabilization procedure is to exclude false positive transformants and also to check the sensitivity of the cells to the polymer. The results showed that the production of PHA by *A. adeninivorans* has a strong negative influence and only the cells with low or zero polymer production can survive. This effect eliminates these yeast for industrial polymer production.

#### Cells with phasin gene

To try and counter loss of production ability during the stabilization of the strains, an additional gene—*phasin*—was expressed. The resulting strains, AAG_thlp and AAG_bktBp, were analysed and the results showed that overexpression of *phasin* gene had a strong positive influence on PHA synthesis. Most of transformants retained the ability to synthesise PHA and the average loss of production was only 12.0 and 17.3% for AAG_thlp and AAG_bktBp respectively (Fig. 1). The highest content of PHB for AAG_thlp #7 was 9.58% of DCW and 6.71% in AAG_bktBp #14 after 96 h cultivation. Based on these results, these transformants were used in further experiments.

### Time-course experiments

Changes of PHA content of the cells are dependent on cultivation time and carbon source. Both factors were explored in simple shake flask experiments without additional feeding. In the first experiment strain AAG_thlp #7 was cultivated in YMM-glc-NO_3_ and YPD medium. After 120 h, the polymer content was 13.3% and 18.7% of DCW and 1.02 and 2.61 g L^−1^ of PHB for YMM and YPD medium respectively. Additionally there was no decrease in polymer level over the cultivation period. Based mainly on the final polymer yield per volume of culture medium, the rich medium was chosen for further experiments.

Strains AAG_thlp #7 and AAG_bktBp #14 were compared in rich media without additional C-sources. The two strains differ only in the type of thiolase they have, either *thl* or *bktB*. After 240 h, the strain with *thl* thiolase reached 26.7% of DCW of PHB compared to 19.8% of DCW for strain with *bktB* thiolase (Table 3). This difference in PHB production was predicted based the findings of Wang et al. [4] and our previous results [24].

**Table 3:** PHA production using different co-substrates

Strain	Co-substrate	PHB-V (% of DCW)	PHB-V (g L^−1^)	PHV (%mol)
AAG_thlp #7	None	26.7	4.62	–
	Glucose	30.9	5.67	–
	Ethanol	42.9	8.35	–
	1-Propanol	28.4	4.63	7.30
	Valeric acid	23.0	2.86	1.86
	Propionic acid	21.1	2.17	1.07
	Sodium Propionate	31.5	4.05	8.38
AAG_bktBp #14	None	19.8	3.16	–
	Glucose	23.8	4.21	–
	Ethanol	30.5	3.48	–
	1-Propanol	20.3	2.87	22.5
	Valeric acid	21.8	2.04	6.32
	Propionic acid	18.6	2.04	3.89
	Sodium propionate	26.9	2.63	16.9
Negative control		0.00	0.00	–

Cultures were incubated for 240 h in rich media (YPD) with the addition of different co-substrates. Negative control—strain transformed with empty plasmid and incubated in the corresponding conditions

### Co-substrates

**Fig. 2:**
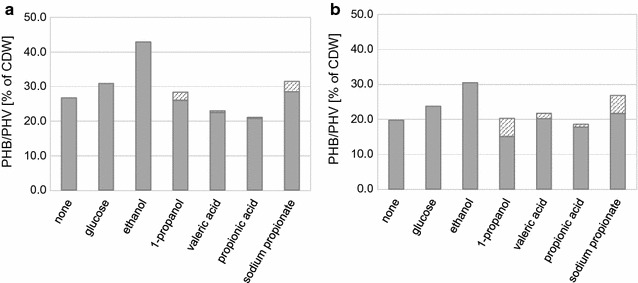
PHB-V *co*-polymer content of cells cultivated on rich media supplemented with different C-sources. **a** AAG_thlp #7; **b** AAG_bktBp #14. The cultures were fed with a C-source after 48/96 h; *grey and shaded fields* represent PHB and PHV fractions respectively

Poly(hydroxyvalerate) was not detected in any of the above experiments and in an attempt to increase PHV synthesis additional substrates were trialled. Four substrates: ethanol, 1-propanol, propionic acid and valeric acid were added at a final concentration of 1% (EtOH, 1-PrOH after 48 h) and 0.1% (PrCOOH, VrCOOH after 48, 72 and 96 h). The results showed that EtOH increases PHB production substantially while a small amount of PHV synthesis occurred when 1-PrOH, PrCOOH and VrCOOH (Table 3; Fig. 2) were added. The maximum level of PHB was obtained for AAG_thlp #7 when ethanol was used (42.9% of DCW of PHB after 240 h of cultivation). On the other hand in the sample with 1-propanol, PHB decreased to 26.0% of DCW but with an additional 2.35% of DCW of PHV. For strain AAG_bktBp #14 the highest content of PHB was also obtained for ethanol (30.5% of DCW), however, because of increased growth on glucose, the highest production was for glucose (4.21 g L^−1^). Use of 1-propanol resulted in cells producing the copolymer (20.3% of DCW) with 22.5%mol of PHV (compared to 7.30%mol for *thl* strain). Use of propionic and valeric acid for both strains gave lower yields and created problems because of their toxicity and difficult handling.

Sodium propionate was also used as a substrate. For this cultivation the concentration of the substrates were calculated so as to be the same molar carbon level which were 0.45, 0.38 and 0.50% for glucose, 1-propanol and sodium propionate respectively. These substrates were added at 48 and 96 h. The results shown in Table 3 revealed a similar *co*-polymer content and PHV contribution for both 1-propanol and sodium propionate. However, when the growth behaviour was compared, cells fed with sodium propionate grew more slowly (sodium propionate is commonly used as a food preservative). Thus, while use of ethanol as a substrate is the best way to increase PHB, 1-propanol increases PHV production.

### Double mutant strains

**Fig. 3:**
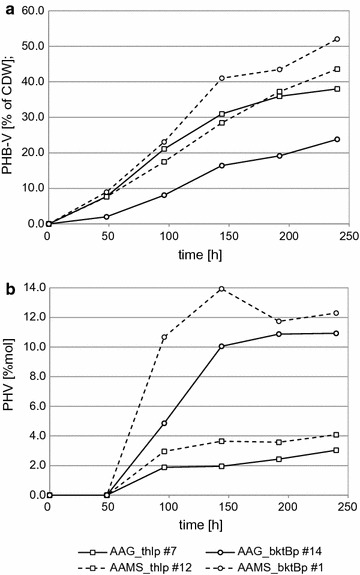
PHB-V *co*-polymer accumulation for single and double transformed strains. **a** PHB-V content; **b** PHV content

Production of polymers by yeast cells can be dependent on several factors, e.g. C-sources, precursor and cofactor availability and growth conditions. Additionally, gene expression levels also may have an influence on PHB-V synthesis. To increase enzyme levels, a double auxotrophic strain *A. adeninivorans* MS1006 [*aleu2 atrp1::ALEU2 aade2::ALEU2*] was transformed twice with the same expression plasmid. The resulting strains, AAMS_thlp and AAMS_bktBp, were tested for PHB-V synthesis using rich media supplemented after 48 and 96 h with ethanol/1-propanol mixture (Fig. 3). After 240 h strain AAMS_bktBp #1 accumulated 52.1% of its DCW as PHB-V (10.8 g L^−1^) of which 12.3%mol was PHV. This was the maximal level of polymer obtained by *A. adeninivorans* in shaking flasks cultures. In the second strain (with *thl* thiolase) 43.6% of its DCW (7.80 g L^−1^) was PHB-V of which 4.08% was PHV. For comparison, strains transformed once with the expression plasmid, had lower levels of productivity; 38.0% DCW was PHB-V (7.67 g L^−1^; 3.04%mol PHV) and 26.8% DCW was PHB-V (4.12 g L^−1^; 10.9%mol PHV) for AAG_thlp #7 and AAG_bktBp #14 respectively (Table 3).

Figure 3b shows the trend of PHV accumulation over the period of the experiment. The %mol of PHV for strain with *thl* thiolase is nearly constant through whole cultivation (except at the first measurement point before EtOH/1-PrOH feeding) while the *bktB* thiolase strain PHV contribution increased after both supplementation points. This demonstrates that using *bktB* thiolase and 1-propanol feeding can increase PHV content in the polymer produced by *A. adeninivorans*.

### Bioreactor results

Optimization of culture conditions for improving polymer synthesis was conducted by a controlled fed-batch fermentation. At first, optimized conditions for growth and protein synthesis (aerobic, glucose and nitrogen feeding) was used with strain AAG_thlp #7. After 52 h cells produced only 0.961% of DCW of PHB (0.981 g L^−1^). In similar conditions that were hypoxic instead of aerobic, production was slightly higher with 4.03% DCW PHB (1.58 g L^−1^) after 111 h (longer period of cultivation is due to the slower consumption of glucose). The next step was to use ethanol as an additional carbon source without nitrogen feeding (the same basic medium). Catabolism of ethanol in yeast is an aerobic process and maximal aerobic conditions in the bioreactor were maintained. After 144 h, strain AAG_thlp #7 was able to accumulate up to 10.7% of DCW of PHB (10.7 g L^−1^). To induce synthesis of the PHV fraction, a mixture of equal volumes of ethanol and 1-propanol was added. The three strains selected for PHB-V production were finally grown on the basic modified rich medium with ethanol/1-propanol, without nitrogen feeding and with pO_2_ at 40%. As expected, PHV was found in all cultivation conditions (Fig. 3). Strain AAG_thlp #7 accumulated 6.95% of DCW of PHB-V (7.36 g L^−1^) after 113 h but with very low PHV contribution (maximum 1.18%mol after 46 h). For both strains with *bktB* thiolase, the results were improved with the highest level obtained by strain AAMS_bktBp #1 with 15.3% of DCW of PHB-V (11.6 g L^−1^) after 113 h growth, compared to 11.0% (10.4 g L^−1^) for strain AAG_bktBp #14 at 113 h. Also PHV concentrations were significantly higher for both strains. At the end of the cultivation, 23.1%mol of PHV was found for AAMS_bktBp #1 in contrast to 21.3%mol of PHV for AAG_bktBp #14.

**Fig. 4:**
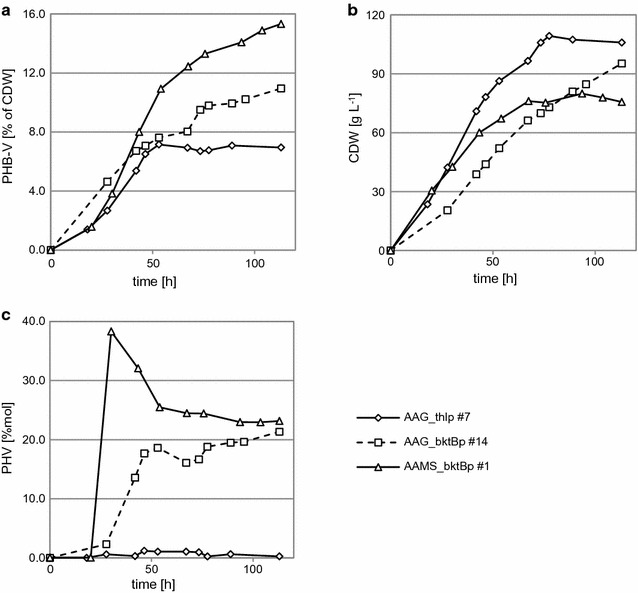
Bioreactor aerobic cultivation results. Cells were cultivated on modified rich medium followed by ethanol/1-propanol (1:1 v/v) feeding: **a** PHB-V content; **b** cell dry mass; **c** PHV fraction contribution

Figure 4c shows that, while the maximum accumulation for AAMS_bktBp #1 was 38.3%mol of PHV after 30 h, the concentration decreased until end of the cultivation. However PHV production in AAG_bktBp #14 rose continuously until the end of the cultivation. Polymer synthesis can have a negative influence on growth and the strain with the lowest polymer production had the highest rate of growth (106 g L^−1^ after 113 h), while in the best PHB-V producer growth rate was the lowest (75.7 g L^−1^ after 113 h).

### Polymer analysis and microscopy imaging

Poly(hydroxybutyrate-*co*-hydroxyvalerate) *co*-polymer produced by strain AAMS_bktBp #1 was extracted using an optimized extraction procedure, which resulted in 71.9% polymer recovery with 99.2% purity.

The extracted material consisted of 73.1%wt of PHB and 26.1%wt of PHV. GPC analysis revealed that M_N_ (number average molar mass) and M_W_ (weight average molar mass) values were 8630 and 17,300 respectively which is considered to be very low. The calculated polydispersity index is 2.00 (M_W_/M_N_). The melting point (*T*_*m*_) and glass transition temperature (*T*_*g*_) were analysed using the DCS method. The first and second *T*_*m*_ was 163.3 and 138.1 °C respectively, which are unexpectedly higher than other PHB-V *co*-polymers with similar PHV fractions [39]. *T*_*g*_ is also higher with a value 55.3 °C compared to −5 °C for a *co*-polymer with 20%mol PHV. Thermogravimetric analysis allows the thermal degradation temperature to be calculated. Degradation of the *co*-polymer started at 260 °C with a sharp decrease of M_W_ at 300 °C with a final loss of 99.2% of the M_W_. These values are similar to other PHB-V *co*-polymers [40]. The TOF-SIMS study confirmed the results obtained by GC/MS, especially the proportion of PHB and PHV. Furthermore, a trace of polydimethylsiloxane was found which probably come from anti-foam agent using to stop foaming during growth in the bioreactor.

**Fig. 5:**
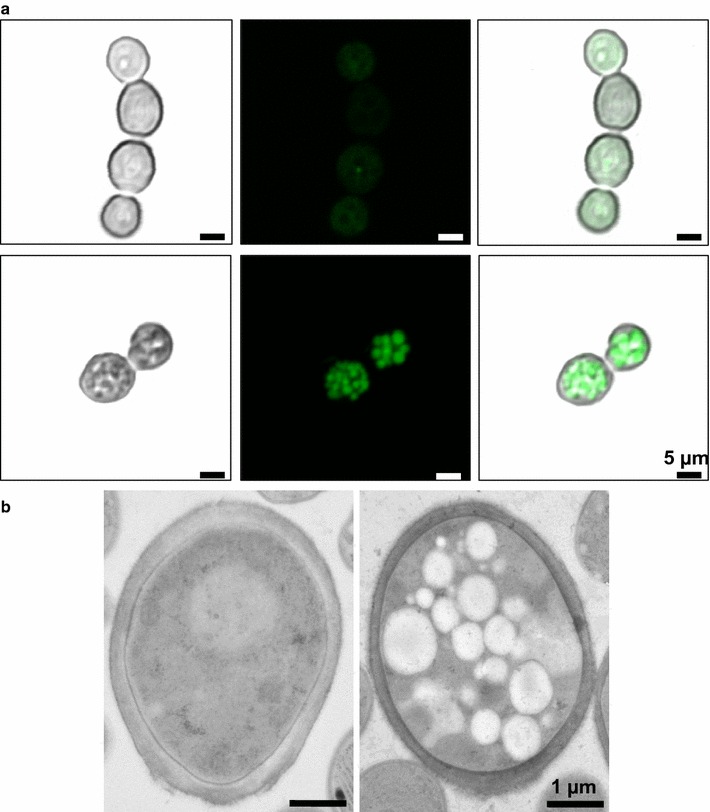
Microscopic analysis of PHB-V inclusions in *A. adeninivorans*: **a** cells have been stained using BODIPY 493/503. *First row*—MS1006 negative control double transformed with empty plasmid; *second row*—AAMS_bktBp #1. The *bars on the right-bottom corner* represent 5 µm. *Left column*—without fluorescence, middle—fluorescence imaging, right—merged images; **b** TEM images. The *bars in the lower right corner* represent 1 µm. Left—negative control, right—AAMS_bktBp #1. *Light spots* represent polymer inclusions

Microscopic analysis of cells using BODIPY 493/503 staining agent was performed. Due to cytoplasmic targeting of PHAs pathway proteins, polymer granules were supposed to be located in cytoplasm. Microscopic images suggested that may be the case (Fig. 5a) and TEM analysis confirmed it (Fig. 5b). PHAs in *Arxula* exist as many granules, in contrast to the few granules seen in bacteria, however, the granule size is similar in yeast and bacteria [17].

## Discussion

The overexpression of the genes, *ß*-*ketothiolase*, *acetoacetyl*-*CoA reductase* and *PHAs synthase* in *A. adeninivorans,* made it able to synthesize the PHB polymer and PHB-V *co*-polymer. The types of *thiolase* and *reductase* genes that were chosen were based on our previous results [24] but another *thiolase*—*bktB* gene was investigated for an increased proportion of the PHV fraction [4]. A lack of an endogenous PHA degradation system allows accumulation of polymer during prolonged incubation experiments and resulted in high levels of polymer in the cell.

*Arxula adeninivorans* was previously used for PHA production but the results were poor [19]. Our initial research showed that lack of stability in the transformants resulted in *A. adeninivorans* losing most of its capacity to synthesise the polymer. This phenomenon may be, in theory, explained by increased cell stress and the inability of cells to multiply. To counter this effect, the *phasin* gene, which is one of the factors for PHA granule stabilization in bacteria [41] and is known to decrease of cell stress in *E. coli* [18], was introduced. Overexpression of the *phasin* gene resulted in the stabilization of the production of the polymer, even during extended cultivation.

Results show that rich media (YPD) is preferable for PHA synthesis, possibly due to higher protein content and the availability of precursors and cofactors. Without co-substrates, *Arxula* is able to produce only PHB and to investigate the development of *co*-polymerization of PHB-V, different C-sources were investigated as co-substrates. Ethanol proved to be the best co-substrate for PHB accumulation and has been shown to be the preferred substrate for synthesis of secondary metabolites in yeast due to its direct conversion to acetyl-CoA in the cytoplasm [42]. Furthermore other co-substrates also allowed *Arxula* to produce the PHB-V *co*-polymer. Although sodium propionate resulted in the highest proportion of PHB-V as a % of cell weight, the toxicity of the molecule reduced the DCW. The use of 1-propanol as the co-substrate gave the highest yield in g L^−1^ which led to its use in all subsequent experiments. Moreover, *Arxula* is able to use C_2_–C_6_ alcohols and convert them to relevant CoA compounds which may theoretically led to synthesis of medium-chain-length PHAs [43]. Thus an equal mixture of ethanol and 1-propanol was chosen to maximise *co*-polymer production.

The strains used in our research differed only in the type of thiolase they contained. The strain with *thl* thiolase was the superior polymer producer, however because the product of this thiolase is acetoacetyl-CoA, which is precursor of the 3HB monomer, the resulting *co*-polymer contained low levels of PHV (up to 8.38%mol). The strain with the *bktB* thiolase, produced β-ketovaleryl-CoA, which although it accumulated at a lower level, had almost 3-times as much PHV. In the strains transformed twice with the same expression plasmid, the strain with *bktB* thiolase accumulated the most PHB-V *co*-polymer. While the PHV fraction was half that in the single transformation strains, it was consistently 3-times higher than in the *thl* thiolase strain. The production of PHB-V to over 50% of DCW for the double transformed strain may be explained by an increase in the enzymes present (which was the aim of double transformation).

In theory, controllable fed-batch fermentation should increase production by for example, better aeration or pH control. However our study on *Arxula* cells gave disparate results. While cells were able to accumulate more than 50% PHB-V of DCW in the shake flasks experiment, growth in the bioreactor resulted in around 15% PHB-V of DCW, however the final polymer level calculated in g L^−1^ was 7.41% higher in the bioreactor experiments. This effect revealed a correlation between growth behaviour and polymer accumulation. Using the same cultivation conditions, strain AAG_thlp #7 accumulated 6.95% DCW of PHB-V with 106 g L^−1^ of total DCW in contrast to 15.3% DCW of PHB-V with 75.7 g L^−1^ of total DCW for AAMS_bktBp #1. Thus the highest production of PHB-V by *Arxula* seems to be a compromise between the level of growth and the accumulation process.

The study of the extracted polymer provided interesting information. Material accumulated by *Arxula* has a low to very low average molecular mass compared to other microbially produced PHAs which are up to 150-times heavier [44]. The low-molecular-weight of the polymer cannot be explained by the size of the granules due to their similarity in yeast and bacteria (Fig. 5).

The low molecular weight has an influence on the other properties. Most unusual is the glass transition temperature, which is around 60 °C higher than that reported for other polymers with similar PHV fractions. This means that products made of the *Arxula* polymer will remain brittle at higher temperature, which is a property that undesirable in some applications, however the melting temperature is higher than usual, making this material more robust at higher temperatures. An additional advantage of a low molecular weight polymer may be faster biodegradation [45]. These differences may come from polydimethylsiloxane, a compound that is present in the polymer, or from other unknown impurities. It is also possible that the extraction method may have an influence on the polymer properties and this needs to be investigated.

## Conclusion

In this paper we have demonstrated that the non-conventional yeast, *A. adeninivorans,* is able to produce 52.1% of DCW of PHB-V (10.8 g L^−1^) with 13.2%mol of PHV in shaking flask and 15.3% of DCW of PHB-V (11.6 g L^−1^) with 23.1%mol of PHV in fed-batch bioreactor cultivation, which are the highest amounts ever seen in yeast. These results show that *A. adeninivorans* could be a useful host organism for the production of PHAs. Future work to increase the production of the polymer will include shifting the flux of acetyl-CoA in direction of PHA synthesis by genetic engineering and further optimization of fed-batch growth conditions and scaling up of production process.

## Abbreviations

PHAs: polyhydroxyalkanoates
PHB: poly(hydroxybutyrate)
PHV: poly(hydroxyvalerate)
PHB-V: poly(hydroxybutyrate-*co*-hydroxyvalerate)
GC/MS: gas chromatography/mass spectrometry
DCE: 1,2-dichloroethane
YRC: yeast rDNA integrative expression cassette
YIC: yeast integrative expression cassette
dcw: dry cell weight
GPC: gas permeation chromatography
DCS: thermoanalysis
TGA: thermogravimetry
TOF-SIMS: time-of-flight secondary ion mass spectrometry
TEM: transmission electron microscopy
PrOH: 1-propanol
PrCOOH: propionic acid
VrCOOH: valeric acid
T_m_: melting temperature
T_g_: glass transition temperature
M_N_: number average molar mass
M_W_: weight average molar mass
